# Presynaptic GABA_B_ receptor–mediated network excitation in the medial prefrontal cortex of Tsc2^+/-^ mice

**DOI:** 10.1007/s00424-021-02576-5

**Published:** 2021-07-19

**Authors:** Davide Bassetti, Heiko J. Luhmann, Sergei Kirischuk

**Affiliations:** Institute of Physiology, University Medical Center of the Johannes Gutenberg University Mainz, Duesbergweg 6, 55128 Mainz, Germany

**Keywords:** Autistic spectrum disorder, E/I ratio, MTOR, Presynaptic tonic inhibition, Hyperexcitability

## Abstract

The TSC1 and TSC2 tumor suppressor genes control the activity of mechanistic target of rapamycin (mTOR) pathway. Elevated activity of this pathway in Tsc2^+/-^ mouse model leads to reduction of postsynaptic GABA_B_ receptor–mediated inhibition and hyperexcitability in the medial prefrontal cortex (mPFC). In this study, we asked whether presynaptic GABA_B_ receptors (GABA_B_Rs) can compensate this shift of hyperexcitability. Experiments were performed in brain slices from adolescent wild-type (WT) and Tsc2^+/-^ mice. Miniature and spontaneous postsynaptic currents (m/sPSCs) were recorded from layer 2/3 pyramidal neurons in mPFC using patch-clamp technique using a Cs^+^-based intrapipette solution. Presynaptic GABA_B_Rs were activated by baclofen (10 µM) or blocked by CGP55845 (1 µM). Independent on genotype, GABA_B_R modulators bidirectionally change miniature excitatory postsynaptic current (mEPSC) frequency by about 10%, indicating presynaptic GABA_B_R-mediated effects on glutamatergic transmission are comparable in both genotypes. In contrast, frequencies of both mIPSCs and sIPCSs were suppressed by baclofen stronger in Tsc2^+/-^ neurons than in WT ones, whereas CGP55845 significantly increased (m/s)IPSC frequencies only in WT cells. Effects of baclofen and CGP55845 on the amplitudes of evoked (e)IPSCs confirmed these observations. These data indicate (1) that GABAergic synapses are inhibited by ambient GABA in WT but not in Tsc2^+/-^ slices, and (2) that baclofen shifts the E/I ratio, determined as the ratio of (m/s)EPSC frequency to (m/s)IPSC frequency, towards excitation only in Tsc2^+/-^ cells. This excitatory presynaptic GABA_B_R-mediated action has to be taken into account for a possible medication of mental disorders using baclofen.

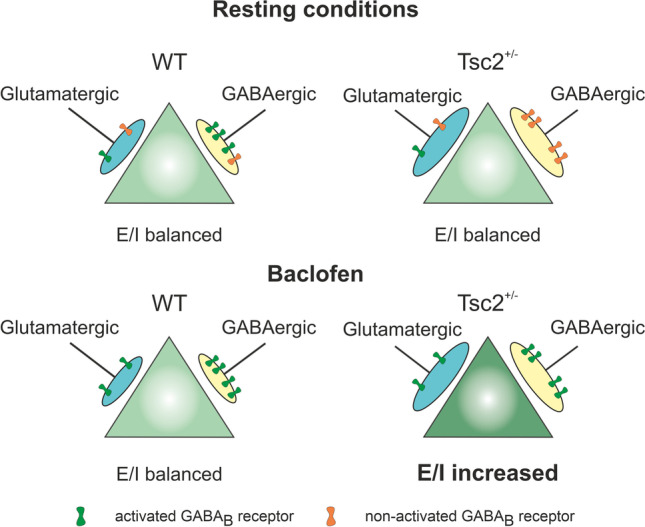

## Introduction

Tuberous sclerosis complex (TSC) is an autosomal dominant disorder that affects many organs, including the brain. Clinical features of TSC vary between individuals and can include brain tumors, seizures, and neurodevelopmental disorders like autism spectrum disorder (ASD) (for recent review [11, 14]). A loss-of-function mutation in one of the tumor suppressor genes, TSC1 (hamartin) or TSC2 (tuberin) underlies TSC [7]. A mutation of either Tsc1 or Tsc2 leads to an enhanced mTOR pathway activity and ultimately to TSC symptoms [9, 13]. The mTOR pathway plays a pivotal role in the regulation of a wide array of cellular functions, including both excitatory and inhibitory synaptic transmission [26]. However, the specific cascades of cellular changes that alter and/or destabilize the neuronal networks in the brain remain elusive. In particular, the sequence of events that could lead to the appearance of ASD symptoms independently of epileptic ones, i.e., in the conditions observed in Tsc2^+/-^ mice, is of particular interest.

It is generally believed that a physiological balance between excitatory and inhibitory synaptic drives in the central nervous system (CNS) is of crucial importance for normal brain development (but see [1]). Even a transient imbalance between excitatory and inhibitory function (E/I ratio) during early development might lead to an immediate or later manifestation of patho(physio)logical symptoms. Conditional deletion of Tsc1 increases the amplitude of miniature excitatory postsynaptic currents (mEPSCs) in hippocampal neurons, suggesting a postsynaptic strengthening of glutamatergic inputs [4, 27]. In contrast to the Tsc1 model, spontaneous Tsc2 mutation (Tsc2^+/-^) appears not to affect basal glutamatergic transmission in rat hippocampus [2, 8, 30]. Similar to the strengthening of excitatory synaptic transmission, the weakening of GABAergic inputs disturbs the E/I balance, and this can contribute to development of neurologic deficits in ASD (for review [12, 22]). Reduced frequency of miniature inhibitory postsynaptic currents (mIPSCs) in hippocampus has been reported in the pan-neuronal Tsc1 knockout mouse model [34]. Stimulation of GABAergic transmission with benzodiazepines, allosteric modulators of GABA_A_ receptors, improves deficits in social interaction in BTBR T + Itpr3tf/J (BTBR) mice [10]. In addition to phasic GABAergic transmission, postsynaptic tonic inhibition can influence neuronal network stability [28]. In our recent study, we have shown that GABA_A_R-mediated postsynaptic inhibition develops by the end of the first postnatal month in the medial prefrontal cortex (mPFC) of both WT and Tsc2^+/-^ cortices. However, its strength has been found to be not significantly different in both genotypes. In contrast, shunting GABA_B_R-mediated postsynaptic inhibition in the mPSC protects neuronal networks against overexcitation induced by a destabilizing factor, for instance kainate, in WT cortices but not in Tsc2^+/-^ ones [3].

Expression of functional GABA_B_Rs in the mPFC has been previously reported [31]. Activation or inhibition of GABA_B_Rs influences the frequency of spontaneous EPSCs and modulates the duration of cortical UP-states [31]. Modulatory action of GABA_B_Rs on the UP-states has been reported in various species and cortex regions [6, 21]. The reported inhibitory action of baclofen, a GABA_B_R agonist, on spontaneous glutamatergic transmission in the mPFC suggests that functional GABA_B_Rs are located on glutamatergic boutons. However, the question of an involvement of GABA_B_Rs on presynaptic GABAergic terminals in mPFC in the modulation of synaptic transmission and in particular in the framework of ASD models has not been extensively investigated yet. Interestingly, higher expression level of presynaptic GABA_B_Rs has been shown immunohistochemically in the mPFC in a rat model of schizophrenia (APO-SUS). Moreover, reduced paired-pulse ratio of evoked IPSCs observed in this model could be corrected by CGP55845, a GABA_B_R antagonist, indicating a contribution of presynaptic GABA_B_Rs located on GABAergic terminals [23]. Changes in GABA_B_R functioning have been also reported in Fmr1-knockout mice, a model of fragile X syndrome. In this model, a selective weakening of GABA_B_R-mediated presynaptic inhibition of glutamatergic synaptic transmission has been reported, while both postsynaptic and presynaptic GABA_B_R-mediated suppression of GABAergic inputs were not modified [15]. As baclofen alleviates behavior deficits only in some, for instance 16p11.2 and BTBP ASD mouse models [24, 25], but not in Fmr1-knockout mice [33], we aimed to investigate the physiological role of presynaptic GABA_B_Rs in the mPFC of Tsc2^+/-^ mouse model.

In this work, we aimed to address the following questions: (1) Are functional presynaptic GABA_B_Rs expressed in both glutamatergic and GABAergic synaptic terminals? (2) Is their functionality dependent on the genotype? (3) How does activation or blockade of GABA_B_Rs affects the E/I balance in the mPFC at the single cell and network level? In order to minimize a potential contribution of postsynaptic GABA_B_Rs, E(I)PSCs were recorded using a Cs^+^-based intrapipette solution. Here, we report that both glutamatergic and GABAergic terminals express functional GABA_B_Rs in both genotypes. In case of glutamatergic contacts, an activation/inhibition of presynaptic GABA_B_Rs suppresses/potentiates glutamatergic synaptic transmission to the same extent in both genotypes. In contrast to glutamate, the GABAergic system demonstrates genotype-dependent sensitivity to GABA_B_R modulators. Baclofen demonstrates a more robust inhibitory effect in Tsc2^+/-^ neurons compared with WT cells, while CGP55845 enhances GABAergic transmission only in WT neurons. These results demonstrate that GABAergic terminals in both genotypes express functional presynaptic GABA_B_Rs. However, the latter are tonically activated by ambient GABA only in WT cortices. Baclofen suppresses the GABAergic system more robustly in the Tsc2^+/-^ mPFC. As a consequence, in Tsc2^+/-^ mice, baclofen treatment shifts the E/I balance towards excitation both at the single cell and at the network level. As baclofen has been suggested as a potential pharmacological treatment of ASD, this excitatory action of baclofen in the mPFC in case of Tsc2 mutation should be taken into account.

## Materials and methods

### Experimental subjects

Animals (B6;129S4-*Tsc2*^*tm1Djk*^/J, [18]) were provided by Jackson Laboratory. Mice were bred and genotyped by the Translation Animal Center (Mainz). Augmented activity of mTOR in Tsc2^+/-^ animals was confirmed by western blot analysis [3]. All experiments were performed in line with the EU directive 86/609/EEC and approved by Landesuntersuchungsanstalt Rheinland-Pfalz (23.177–07/G 10–1-010). All experiments were designed to minimize the number of animals used.

### Brain slice preparation

Wild-type (WT) and Tsc2^+/-^ littermates of postnatal day (P) 25–40 were used for experiments. Brain slice preparation procedure was described elsewhere [3]. Briefly, animals were deeply anesthetized using isoflurane and decapitated. The brain was taken out and submerged in protective artificial cerebrospinal fluid (ACSF), which contained (in mM): 110 choline chloride, 2.5 KCl, 1.25 NaH_2_PO_4_, 25 NaHCO_3_, 20 glucose, 11.6 sodium l-ascorbate, 3.1 sodium pyruvate, 0.2 CaCl_2_, and 5 MgCl_2_. ACSF was continuously bubbled with a 95% O_2_ and 5% CO_2_ gas mixture, to set the pH value. For brain extraction, ACSF was cooled down to zero degrees (°C) using ice. Coronal slices were sectioned at 300 µm using a vibratome (Campden Instruments Ltd., Loughborough, UK). Slices containing the medial prefrontal cortex (mPFC) were selected using the mouse brain atlas [20]. Before recording, slices were allowed to recover in ACSF at room temperature for at least 1 h. For recovery and recording, the ACSF contained (in mM) 125 NaCl, 2.5 KCl, 10 glucose, 1.25 NaH_2_PO_4_, 25 NaHCO_3_, 2CaCl_2_, and 1 MgCl_2_, and was continuously oxygenated with a 95% O_2_ and 5% CO_2_ gas mixture.

### Whole-cell patch-clamp recordings

For electrophysiological recordings, brain slices were transferred into a recording chamber on the microscope stage (Axioscope FS, Zeiss, Germany). Slices were kept continuously perfused with ACSF via a gravity-driven system. The flow rate was set to about 1.5 ml/min. The recording chamber volume was about 0.7 ml. The temperature was kept at 31–32 °C using a temperature-controller (Temperaturcontroller III, Luigs & Neumann, Ratingen, Germany).

Firstly, slices were visually inspected using a × 5 objective prior to any experiment. After this, a × 40 objective (Zeiss, Oberkochen, Germany) was used to identify and select layer 2/3 pyramidal neurons according to morphological criteria. Pipettes were pulled from borosilicate glass capillaries using a P-87 horizontal puller (Sutter Instrument Co., Novato, USA). Their resistance amounted to 3–6 MOhm when filled with intrapipette solution. The latter contained (in mM) 125 CsOH, 125 gluconic acid, 5 CsCl, 10 EGTA, 10 HEPES, 2 MgCl_2_, 2 Na-ATP, and 0.4 Na-GTP. pH was adjusted to 7.3 using CsOH. Osmolarity was measured and set to 320 mOsm using sucrose.

Acquisition of postsynaptic currents (PSCs) was performed using an EPC-10 amplifier using the TIDA 5.24 software (HEKA Elektronik, Lambrecht, Germany). The liquid junction potential was not corrected. Recorded signals were filtered at 3 kHz and digitally sampled at 10 kHz. After successful establishment of whole-cell configuration, hyperpolarizing pulses of 10 mV were applied to enable an estimation of passive properties of the cell such as cell capacity, access resistance, and series resistance. Series resistance compensation was not applied. Recordings with series resistance higher than 30 MOhm were discarded.

Synaptic currents were recorded in voltage clamp mode. In particular, we recorded spontaneous postsynaptic currents (sPSCs) and miniature postsynaptic currents (mPSCs) in either standard ACSF (sPSCs) or in the presence of 0.5 µM tetrodotoxin (TTX), a blocker of voltage-gated sodium channels (mPSCs). Excitatory postsynaptic currents (s/m)EPSCs were recorded at a holding potential of − 70 mV, the reversal potential for inhibitory postsynaptic currents (IPSCs). In order to acquire (s/m)IPSCs, the holding potential was set to 0 mV, the reversal potential for EPSCs.

Evoked inhibitory postsynaptic currents (eIPSCs) were recorded in the presence of DNQX and APV, the antagonists of AMPA and NMDA receptors, respectively. eIPSCs were elicited using focal electrical stimulation in the vicinity of the patched cell (about 100 µm) via a glass pipette filled with ACSF. A custom-made stimulation unit was used to provide rectangular current pulses. The intensity level was tuned to activate a unitary synaptic input (minimal stimulation, as previously described in [29]). Briefly, inputs were located by slowly moving the stimulation pipette in the vicinity of the cell patched, while a train of four pulses was applied every 4 s. After the input was revealed, stimulation intensity was set about 20% higher than the threshold strength for the identified input. Only traces with stable latency between the stimulation artifact and the evoked response were kept for further analysis. The applied stimulus intensity amounted to 1–2 µA. At least 20 responses were recorded in each cell.

### Chemicals

DL-APV, DNQX, and CGP55845 were obtained from Biotrend (Cologne, Germany). All other chemicals were provided from Tocris (Bio-Techne, Wiesbaden, Germany). Aliquots of DNQX and CGP55845 were prepared using dimethylsufoxide (DMSO). All final solutions were prepared shortly before the experiments. Final concentration of DMSO in ACSF was always less than 0.2%.

### Data evaluation and statistics

Evoked IPSCs were analyzed using TIDA 5.24 (HEKA Elektronik, Lambrecht, Germany). mPSCs and sPSCs were evaluated using the PeakCount software. The latter utilizes a derivative threshold-crossing algorithm to detect individual PSCs. All detected PSCs were displayed for visual inspection. Data in text and figures are expressed in mean ± SEM. Statistical analysis was performed using GraphPad Prism 5 (GraphPad Software, Inc., San Diego, USA). Differences between means were tested for significance using one-way ANOVA. To test the difference from a hypothetical mean, one sample Student’s *t*-test was used. Paired values were compared using paired Student’s *t*-test. Statistical significance was presented using the following rules: *p < 0.05, **p < 0.005, ***p < 0.001, ns—not significant.

## Results

### Functional GABA_B_Rs are located on both glutamatergic and GABAergic presynaptic terminals

In the mPFC, functional GABA_B_Rs have been reported to be expressed postsynaptically and presynaptically on glutamatergic boutons [31]. As postsynaptic GABA_B_Rs are tonically activated in WT pyramidal neurons, but not in Tsc2^+/-^ cells, the membrane resistance of WT pyramidal neurons around the end of the first postnatal month is significantly lower [3]. To reduce the effect of persistent postsynaptic GABA_B_R-mediated shunting, whole-cell patch-clamp recordings were performed using a Cs^2+^-based intrapipette solution to block K^+^ conductance (see the “Methods” section). In this case membrane resistances in WT and Tsc2^+/-^ cells were not significantly different (92 ± 3 and 91 ± 4 MOhm, n = 27 and 26 neurons for WT and Tsc2^+/-^, respectively, p = 0.94, F = 0.005). Neither baclofen (10 µM), a GABA_B_R agonist (− 1.5 ± 1.4 and − 1.7 ± 1.5 pA, n = 17 and 15 for WT and Tsc2^+/-^, p = 0.97, F = 0.001), nor CGP55845 (1 µM), a GABA_B_R antagonist (− 0.2 ± 1.5 and − 0.4 ± 2.1 pA, n = 12 and 14 for WT and Tsc2^+/-^, p = 0.99, F = 0.0001, data not shown), could significantly affect the holding current in both genotypes. Furthermore, neither baclofen nor CGP55845 influenced the mean amplitude of either mEPSCs or mIPSCs (Table 1), confirming that postsynaptic GABA_B_Rs mediate their effects mostly via postsynaptic K^+^ channels (GIRKs) which are blocked by intracellular Cs^+^ ions in this study.

**Table 1:** GABA_B_R modulators do not affect mean mEPSC or mIPSC amplitudes

	Control	Baclofen	CGP55845	ANOVA
mEPSC (pA)				
WT	7.5 ± 0.3 n = 14	7.3 ± 0.4 n = 7	7.3 ± 0.3 n = 8	F = 0.18 p = 0.84
Tsc2^+/-^	7.7 ± 0.4 n = 14	7.2 ± 0.9 n = 7	7.2 ± 0.4 n = 7	F = 0.47 p = 0.63
mIPSC (pA)				
WT	9.6 ± 0.5 n = 14	9.2 ± 0.9 n = 7	9.4 ± 0.8 n = 8	F = 0.11 p = 0.89
Tsc2^+/-^	9.2 ± 0.9 n = 14	9.6 ± 1.2 n = 7	9.2 ± 1.1 n = 7	F = 0.05 p = 0.96

**Fig. 1:**
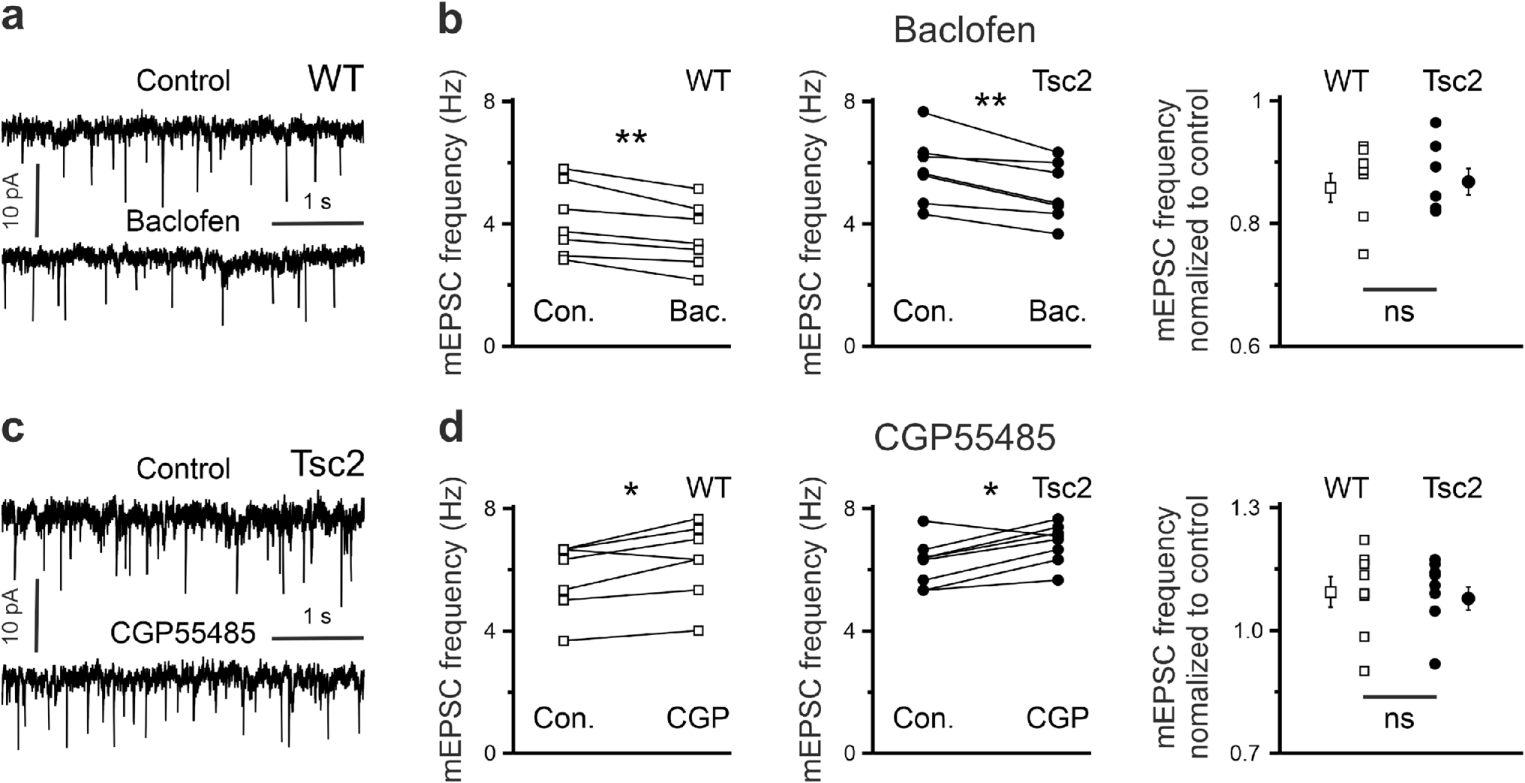
GABA_B_R agonist-induced and antagonist-induced effects on mEPSC frequency do not depend on genotype. **a** Representative mEPSC traces recorded from a WT neuron demonstrate the baclofen-induced effect. **b** Baclofen (10 µM) significantly reduces mEPSC frequency both in WT (left) and in Tsc2^+/-^ (middle) neurons. Relative baclofen-induced reductions of mEPSC frequency are similar in WT and Tsc2^+/-^ cells (right panel). **c** Representative mEPSC traces recorded from Tsc2^+/-^ cells demonstrate the CGP55845-induced effect. **d** CGP55845 (1 µM) significantly increases mEPSC frequency both in WT (left) and in Tsc2^+/-^ (middle) cells. Relative CGP55845-induced elevations of mEPSC frequency are comparable in WT and Tsc2^+/-^ neurons (right panel)

Having confirmed that the target cell is not sensitive to GABA_B_R modulators, we investigated excitatory/inhibitory postsynaptic currents and their sensitivity to baclofen and CGP55845. In the first set of experiments, we recorded mPSCs in the presence of TTX (0.5 µM), an antagonist of voltage-gated Na^+^ channels. In line with our previous work [3], Tsc2^+/-^ neurons at this age displayed significantly increased frequencies of both mIPSCs (4.3 ± 0.4, n = 14, and 5.8 ± 0.3 Hz, n = 14, in WT and Tsc2^+/-^ neurons, respectively, p = 0.004) and mEPSCs (4.1 ± 0.3, n = 14, and 5.3 ± 0.3 Hz, n = 14, in WT and Tsc2^+/-^ neurons, respectively, p = 0.011). Baclofen (10 µM) reduced mEPSC frequencies both in WT (0.87 ± 0.02, n = 7) and in Tsc2^+/-^ (0.86 ± 0.03, n = 7) cells without any significant difference between the genotypes (p = 0.92, F = 0.01, Fig. 1a, b). Blockade of GABA_B_Rs with CGP55845 (1 µM) induced a slight increase in mEPSC frequency in WT (1.11 ± 0.04, n = 8) and Tsc2^+/-^ (1.09 ± 0.03, n = 7) neurons. Also, these values did not differ in a statistically significant way (F = 0.05, p = 0.94, Fig. 1c, d).

**Fig. 2:**
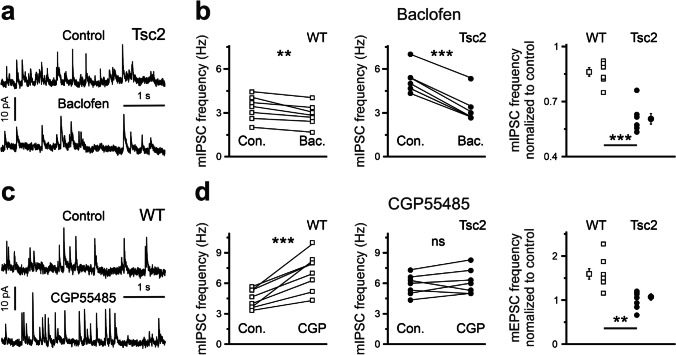
GABA_B_R agonist-induced and antagonist-induced effects on mIPSC frequency differ in WT and Tscs2 ± cells. **a** Representative mIPSC traces recorded from a Tsc2^+/-^ neuron demonstrate the baclofen-induced effect. **b** Baclofen (10 µM) significantly reduces mIPSC frequency both in WT (left) and in Tsc2^+/-^ (middle) neurons. Relative baclofen-induced reduction of mIPSC frequency is significantly larger in Tsc2^+/-^ cells compared with that in WT ones (right panel). **c** Representative mIPSC traces recorded from a WT cell demonstrate the CGP55845-induced effect. **d** CGP55845 (1 µM) significantly increases mIPSC frequency in WT (left) but not in Tsc2^+/-^ (middle) neurons. Relative CGP55845-induced elevation of mEPSC frequency is significantly stronger in WT neurons compared with that in Tsc2^+/-^ cells (right panel)

In contrast to mEPSCs, GABAergic mIPSCs demonstrated a genotype-dependent sensitivity to GABA_B_R modulators. Baclofen decreased mIPSC frequency in WT cells (0.86 ± 0.03, n = 7) and in Tsc2^+/-^ neurons (0.61 ± 0.03, n = 7). This difference was statistically significant (p < 0.0001, F = 47.1, Fig. 2a, b). CGP55845 strongly increased mIPSC frequency in WT neurons (1.59 ± 0.12, n = 8) but only slightly elevated mIPSC frequency in Tsc2^+/-^ cells (1.07 ± 0.05, n = 7). The difference between genotypes was statistically significant (F = 14.4, p = 0.0022, Fig. 2c, d), indicating stronger tonic GABA_B_R-mediated inhibition of synaptic GABA release in WT neurons.

### Evoked IPSCs confirm presynaptic location of GABA_B_Rs

**Fig. 3:**
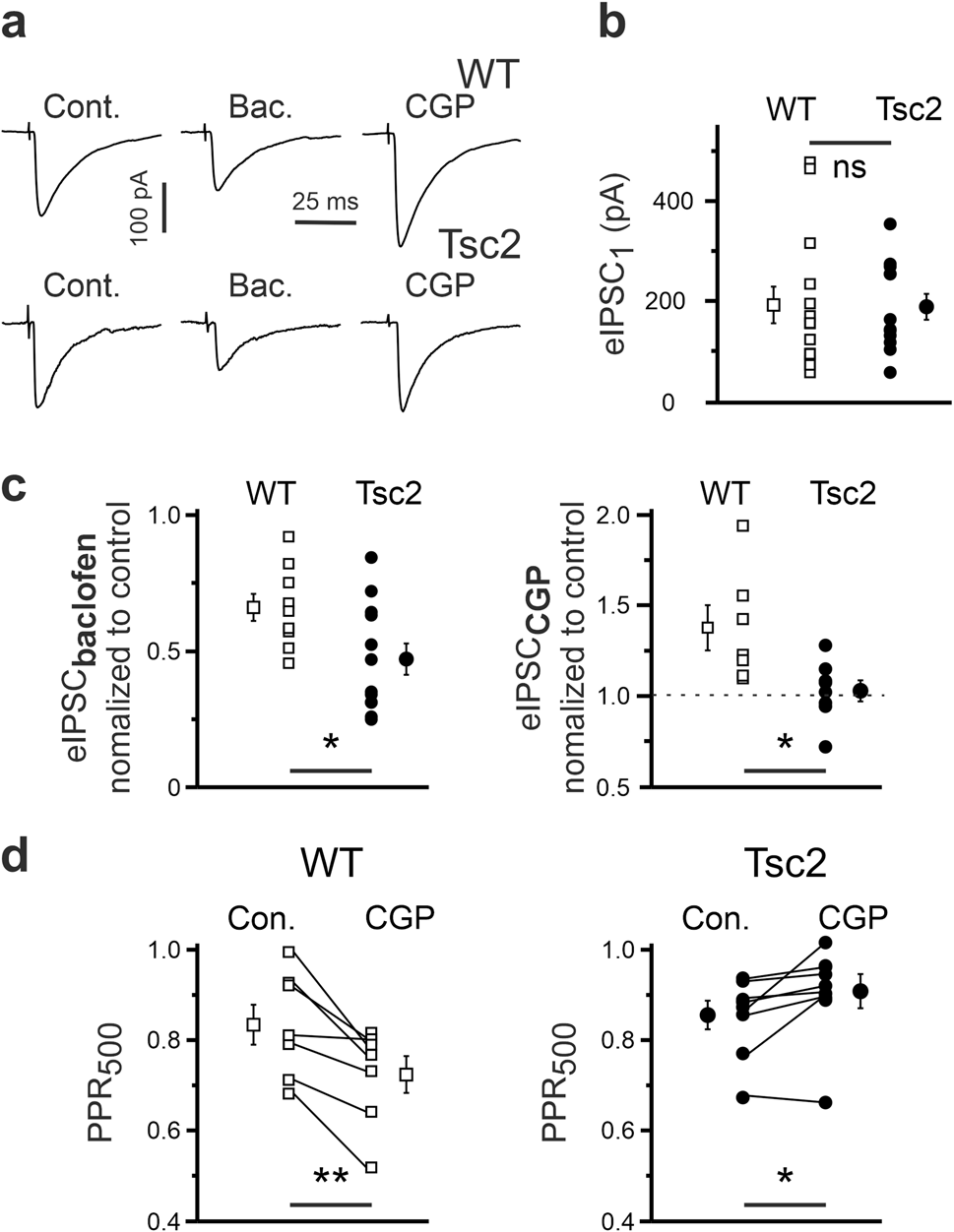
GABA_B_R agonist-induced and antagonist-induced effects on eIPSCs. **a** Representative eIPSC traces recorded from a WT (top) and Tsc2^+/-^ (bottom) cell in control and in the presence of either baclofen (10 µM) or CGP55845 (1 µM). Traces are an average of 20 consecutive recordings. **b** Mean eIPSC amplitudes are not significantly different in WT and Tsc2^+/-^ neurons. **c** Baclofen reduces the mean eIPSC amplitude stronger in Tcs2 ± cells compared with WT neurons (left), whereas CGP55845 increases the mean eIPSC amplitude in WT cells but not in Tsc2^+/-^ neurons (right panel). **d** In the presence of CGP55845 (CGP), the paired-pulse ratio at 500 ms interstimulus interval (PPR_500_) is reduced in WT neurons (left) but increased in Tsc2^+/-^ ones (right panel)

The above data obtained using CGP55845 suggests that presynaptic GABA_B_Rs on GABAergic terminals in WT cortices are tonically activated under control condition. To corroborate this result, we recorded evoked (e)IPSCs using a minimal stimulation approach (see the “Methods” section). Presumably due to the variability of inhibitory inputs (with amplitude ranging from about 80 to 400 pA), the mean eIPSC amplitudes did not differ in the two genotypes (192 ± 37 vs 188 ± 26 pA, n = 14 and 12 for WT and Tsc2^+/-^, respectively, p = 0.94, F = 0.007, Fig. 3a, b). However, GABAergic inputs demonstrated a different sensitivity to GABA_B_R modulators in WT and Tsc2^+/-^ slices. Baclofen reduced the mean eIPSC amplitude stronger in Tsc2^+/-^ cells (0.49 ± 0.06, n = 12) compared with WT (0.66 ± 0.05, n = 9, p = 0.027, F = 5.66) neurons. CGP55845 increased the mean eIPSC amplitude in WT cells (1.38 ± 0.12, n = 7) in a more pronounced way than in Tsc2^+/-^ neurons (1.03 ± 0.06, n = 8, p = 0.02, F = 7.01, Fig. 3a, c). Next, we applied two consecutive pulses separated by 500 ms interstimulus interval (ISI) and calculated the paired-pulse ratio (PPR_500_). The latter was defined as the ratio of the mean amplitude of the second eIPSCs to the mean amplitude of the first eIPSCs. If presynaptic GABA_B_Rs in WT cortices are tonically activated, their blockade with CGP55845 should decrease the PPR_500_. Indeed, in the presence of CGP55845, PPR_500_ was significantly smaller compared with control conditions (0.83 ± 0.04 and 0.72 ± 0.04 in control and in the presence of CGP55845, respectively, n = 7, t = 4.22, p = 0.006, paired Student’s test), thus confirming our hypothesis. In contrast to WT neurons, in Tsc2^+/-^ cells CGP55845 increased PPR_500_ to 0.91 ± 0.04 from 0.86 ± 0.03 in control (n = 8, t =  − 2.9, p = 0.022, paired Student’s test, Fig. 3d). As CGP55845 failed to affect the mean amplitude of the first eIPSCs in Tsc2^+/-^ neurons (Fig. 3c), this data suggests that GABA released by the first stimulus inhibits the second release in an autocrine manner.

### Presynaptic GABA_B_Rs’ effects on the E/I ratio at a single cell level depend on genotype

**Fig. 4:**
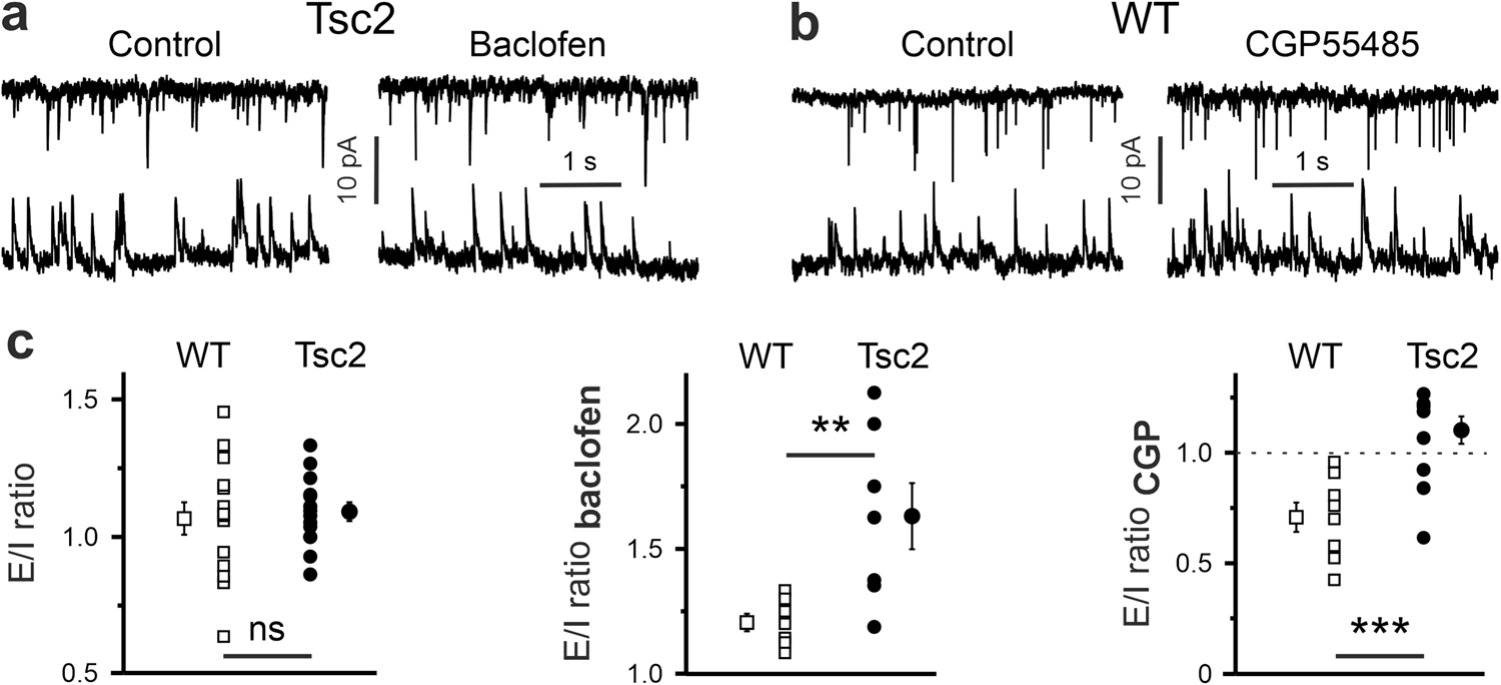
GABA_B_R agonist-induced and antagonist-induced effects on the E/I ratio at a single cell level. **a** Representative mEPSC (top) and mIPSC (bottom) traces recorded from the same Tsc2^+/-^ neuron in control (left) and in the presence of baclofen (10 µM, right panel). **b** Representative mEPSC (top) and mIPSC (bottom) traces of recorded from the same WT cell in control (left) and in the presence of CGP55845 (1 µM, CGP, right panel). **c** Statistical data shows that the E/I ratios are comparable in WT and Tsc2^+/-^ neurons in control (left). The baclofen-induced increase in the E/I ratio is more pronounced in Tsc2^+/-^ cells than in WT cells (middle). CGP55845 decreases the E/I ratio significantly stronger in WT neurons compared with Tsc2^+/-^ cells (right panel)

The above data show that glutamatergic terminals demonstrate comparable sensitivity to GABA_B_R modulators in both genotypes, while this is not the case at GABAergic synapses. Next, we defined the E/I ratio as the ratio of mEPSC frequency to the mIPSC frequency and ask whether GABA_B_R modulators affect this ratio in a genotype-dependent manner. Figure 4 shows that the E/I ratios under control conditions were comparable in WT (1.1 ± 0.1, n = 14) and in Tsc2^+/-^ neurons (1.09 ± 0.03, n = 14, p = 0.71, F = 0.13). Baclofen increased the E/I ratio only slightly in WT cells (1.19 ± 0.04, n = 7), but caused a significantly larger shift towards excitation in Tsc2^+/-^ neurons (1.63 ± 0.13, n = 7). This difference between the two genotypes was statistically significant (n = 17 in both cases, p = 0.009, F = 9.68, Fig. 4a, c). CGP55845 failed to influence the E/I ratio in Tsc2^+/-^ neurons (1.1 ± 0.06, n = 7), but moved the E/I ratio towards inhibition in WT cells (0.71 ± 0.7, n = 7). The difference between the genotypes was statistically significant (p = 0.0008, F = 18.5, Fig. 4b, c). Thus, activation of presynaptic GABA_B_Rs results in a shift towards excitation in Tsc2^+/-^ slices. In contrast, in WT slices, the baclofen-induced effect on the E/I ratio is relatively small, whereas CGP55845 causes a robust change in the opposite direction (i.e., towards inhibition).

### GABA_B_R-mediated effects on the network E/I ratio depend on genotype

**Fig. 5:**
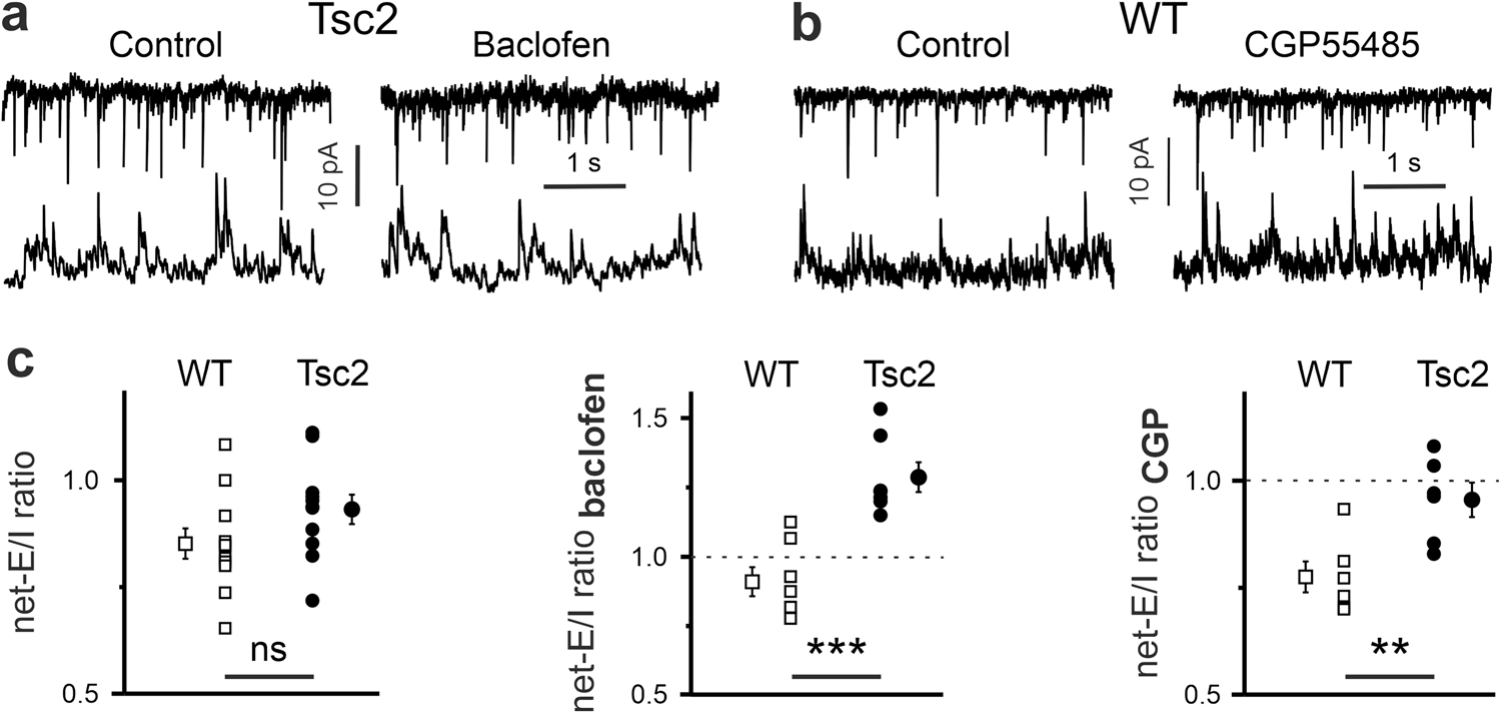
GABA_B_R agonist-induced and antagonist-induced effects on network (net-)E/I ratio. **a** Representative sEPSC (top) and sIPSC (bottom) traces recorded from the same Tsc2^+/-^ neuron in control (left) and in the presence of baclofen (10 µM, right panel). **b** Representative sEPSC (top) and sIPSC (bottom) traces of recorded from the same WT neuron in control (left) and in the presence of CGP55845 (1 µM, right panel). **c** Statistical data shows that the net-E/I ratios are comparable in WT and Tsc2^+/-^ neurons in control (left). The baclofen-induced increase in the net-E/I ratio is more pronounced in Tsc2^+/-^ cells than that in WT cells (middle). CGP55845 decreases the net-E/I ratio significantly stronger in WT neurons compared with Tsc2^+/-^ cells (right panel)

As GABA_B_Rs in mPFC are expressed both presynaptically and postsynaptically, we asked what could be the effect of GABA_B_R modulation on the network (net-)E/I ratio. Using Cs^2+^-based intrapipette solution and standard ASCF, i.e., without TTX added, we recorded alternatively spontaneous (s)EPSCs and (s)IPSCs from the same cell (Fig. 5). In this configuration, both presynaptic and postsynaptic GABA_B_R will be activated/blocked by baclofen/CGP55845 in the whole slice with exception of the patched cell. In this case, we defined the net-E/I ratio as a ratio of sEPSC frequency to sIPSC frequency. Under control conditions the net-E/I ratios were comparable in WT (0.93 ± 0.03, n = 11) and in Tsc2 (0.85 ± 0.04, n = 11, p = 0.12, F = 2.63). Baclofen failed to affect the net-E/I ratio in WT cells (0.91 ± 0.05, n = 7), but shifted it towards excitation in Tsc2^+/-^ neurons (1.29 ± 0.05, n = 7). This difference was statistically significant (p = 0.001, F = 22.6). CGP55845 reduced the net-E/I ratio in WT neurons (0.77 ± 0.04, n = 6), but only slightly influenced the net-E/I ratio in Tsc2^+/-^ cells (0.96 ± 0.04, n = 7). This difference between the genotypes was statistically significant (p = 0.008, F = 11.1). Thus, on the network level, neither activation nor inhibition of GABA_B_Rs shifts the net-E/I ratio towards excitation in WT animals. In contrast to WT slices, GABA_B_R activation strongly potentiates neuronal activity in Tsc2^+/-^ cortices.

## Discussion

In this study, we used whole-cell patch-clamp recordings to investigate synaptic function in layer 2/3 pyramidal neurons in the mPFC in acute brain slices obtained from adolescent WT and Tsc2^+/-^ mice. In our previous work, we have shown that by the end of the first postnatal month, tonically activated GABA_B_Rs mediate postsynaptic shunting inhibition in WT neurons, but not in Tsc2^+/-^ cells, leading to hyperexcitability of mPFC in Ts2 ± animals [3].

In this study, we show that (1) functional presynaptic GABA_B_Rs are present in both excitatory and inhibitory terminals in the mPFC of both WT and Tsc2^+/-^ neurons; (2) in the case of glutamatergic synaptic transmission, GABA_B_R modulations produce comparable effects in the two genotypes; (3) modulation of GABA_B_Rs exerts different effects on GABAergic transmission in the two genotypes. In fact, blockade of GABA_B_Rs disinhibited GABAergic transmission in WT neurons, but not in Tsc2^+/-^ ones, indicating a tonic GABA_B_R-mediated inhibition of GABA release by ambient GABA in WT slices. On the other hand, activation of GABA_B_Rs suppressed GABAergic activity in Tsc2^+/-^ cells much stronger than that in WT neurons, showing that the decreased tonic activation of presynaptic GABA_B_Rs in Tsc2^+/-^ neurons is not necessarily mediated by their decreased functionality. Furthermore, (4) the GABA_B_R modulation effects on the network activity differ between the genotypes. Unexpectedly, activation of GABA_B_Rs shifts the E/I balance towards excitation in Tsc2^+/-^ mice. Since GABA_B_Rs can be activated by ambient GABA, both elevation and decrease of extracellular GABA concentration hardly affect the E/I balance in WT mPFC, while this balancing effect of post-/presynaptic GABA_B_R-mediated inhibition is compromised in Tsc2^+/-^ mPFC.

### Presynaptic GABA_B_Rs in the mPFC

Expression of functional presynaptic GABA_B_Rs at glutamatergic terminals and postsynaptically in pyramidal neurons has been reported in rat mPFC [31]. Baclofen and CGP55432 can decrease and increase sEPSC frequency in pyramidal neurons, respectively. However, as the experiments were performed using a K^+^-based intrapipette solution, the involvement of postsynaptic GABA_B_Rs cannot be completely excluded. In addition, a previous study reported that GABA_B_R modulators affect the duration of UP-states and DOWN-states in the mPFC [31]. Using a genetic approach, an involvement of presynaptic GABA_B_Rs in regulation of UP-states and DOWN-states has been directly shown in mouse enthorinal cortex [6]. Instead of genetic ablation of presynaptic GABA_B_Rs, we used a Cs^+^-based intrapipette solution to block postsynaptic K^+^ conductance and in turn, the effects of postsynaptic GABA_B_Rs. As both baclofen and CGP55845 failed to influence the holding currents, membrane resistance and the mean amplitudes of postsynaptic currents, we assume a minimal influence of postsynaptic GABA_B_Rs on the parameters measured in this work.

### Glutamatergic synaptic transmission

In the first set of experiments, we investigated the effect of modulation of GABA_B_Rs activity on glutamatergic synaptic transmission. The presynaptic location of GABA_B_Rs to glutamatergic synapses in the mPFC has been previously reported in mice [5] and rats [31]. In the rat mPFC, both baclofen and CGP55845 produced significant effects on sEPSCs. This is in line with the data obtained in this study. Both baclofen and CGP55845 significantly affected the frequency of mEPSCs both in WT and in Tsc2^+/-^ cells (Fig. 1), confirming that functional GABA_B_Rs are located on glutamatergic synaptic terminals. The magnitude of GABA_B_R modulator-induced changes of mEPSC frequencies in WT neurons amounted to about 10% in all cases, which is comparable with data reported in [31]. We failed to observe any significant difference between Tsc2^+/-^ and WT cells. As the CGP55845-induced elevation of mEPSC frequency is significant in both genotypes, presynaptic GABA_B_Rs on glutamatergic boutons are tonically activated by ambient GABA in both cases. We conclude that glutamatergic terminals both in WT and in Tsc2^+/-^ mPFC express functional GABA_B_Rs, and the GABA_B_R-mediated effects are comparable in the two genotypes.

### GABAergic synaptic transmission

Although the (patho-)physiological role of presynaptic GABA_B_Rs located on GABAergic terminals is still elusive, their possible involvement in the manifestation of neuropsychological symptoms has been postulated. In this work, we report that GABAergic synaptic terminals are sensitive to GABA_B_R modulators both in WT and in Tsc2^+/-^ cortices, confirming that functional GABA_B_Rs are located on presynaptic GABAergic boutons in the mPFC. However, the strength of a modulator-induced change depends on the genotype. Stimulation of GABA_B_Rs with baclofen decreased mIPSC frequency and the mean eIPCS amplitude significantly stronger in Tsc2^+/-^ neurons compared to WT ones, while the blockade of GABA_B_Rs increased mIPSC frequency and the mean eIPSC amplitude in WT cells, but not in Tsc2^+/-^ ones (Figs. 2 and 3). The latter result shows that GABA release from GABAergic terminals is tonically inhibited by ambient GABA in WT cells, but not in Tsc2^+/-^ neurons. Interestingly, GABA_B_R modulators differently affected PPR of eIPSCs in WT and Tsc2^+/-^ neurons. In the WT cells, CGP55845 strongly increased the mean amplitude of the first eIPSCs and decreased PPR, indicating that presynaptic GABA release probability at WT terminals is tonically suppressed by ambient GABA. In contrast, in the Tsc2^+/-^ neurons, CGP55845 did not affect the mean amplitude of the first eIPSCs, but significantly increased PPR at 500 ms ISI, indicating an autocrine GABA_B_R-mediated inhibition of presynaptic GABA release.

Our results indicate that the tonic activation of inhibitory and excitatory synapses via GABA_B_Rs is dependent on the type of synapse. A potential explanation could lie in a different local extracellular GABA concentration at excitatory versus inhibitory synapses. We suggest an involvement of GABA transporters (GATs), which level was found to be increased in the amygdala of mice exposed to valproic acid, in conjunction with a reduction in GAD67 [17].

### E/I ratio at a single cell level

The observed differences in GABA_B_R-mediated effects on excitatory and inhibitory transmission raise the question of whether presynaptic GABA_B_Rs can influence the E/I balance in the mPFC. As we recorded mEPSCs and mIPSCs from the same cell, it is possible to calculate both the E/I ratio and its sensitivity to GABA_B_R modulators in individual neurons. Since both mEPSC and mIPSC frequencies were increased in Tsc2^+/-^ cells compared with that in WT neurons, the E/I ratio was not significantly different in the investigated age group (Fig. 4 and [3]). As CGP55845 hardly affected mIPSC frequency in Tsc2^+/-^ cells, it only slightly shifted the E/I ratio. In contrast, the GABA_B_R blockade strongly reduced the E/I ratio in WT neurons, i.e., shifted it towards inhibition. Given the activation of GABA_B_Rs had a strong inhibitory effect on mIPSC frequency in Tsc2^+/-^ neurons, baclofen also caused a marked increase in E/I ratio (Fig. 4c). Surprisingly, in WT cells, baclofen also significantly elevated the E/I ratio, although to a smaller extent (~ 20% versus ~ 60% in WT and Tsc2^+/-^ neurons, respectively). Activation of GABA_B_Rs, a Gi/o coupled receptor [19], is traditionally believed to mediate inhibition [16, 32] and a deficit of GABA_B_R-mediated inhibition of glutamatergic inputs can lead to hyperactivity in the cortex [15]. However, in the Tsc2^+/-^ mouse model, presynaptic GABA_B_Rs on GABAergic synaptic terminals appear to be not activated by ambient GABA at resting conditions, resulting in higher GABA release probability which is in line with the increased mIPSC frequency recorded. Baclofen or physiologically elevated extracellular GABA concentration can activate them and reduce the phasic inhibitory drive, leading to increased neuronal activity in the mPFC.

### E/I ratio on the network level

The above conclusion is valid at a single cell level. As mEPSCs/mIPSCs were recorded in the presence of TTX, a blocker of voltage-gated Na^+^ channels, and using a Cs^+^-based intrapipette solution, the influence of postsynaptic GABA_B_Rs on cell excitability is probably negligible. On the other hand, usage of Cs^+^-based intrapipette solution but without TTX added to ACSF would block postsynaptic GABA_B_Rs only in the patched neuron, but not in rest of the slice. Recordings of sEPSCs and sIPSCs would then report the effects of GABA_B_R modulators on the complete neuronal network. As both sEPSCs and sIPSCs were acquired from the same cell, the net-E/I ratio can be calculated. Figure 5 and [3] show that the control net-E/I ratios in WT and Tsc2^+/-^ slices were not significantly different at P25–40. As effects of CGP55845 on both postsynaptic and presynaptic GABA_B_Rs on Tsc2^+/-^ neurons were rather minimal, GABA_B_R blockade produced minimal effects on the net-E/I ratio in Tsc2^+/-^ genotype. Although CGP55845 blocked tonically activated postsynaptic GABA_B_Rs in WT slices, disinhibition of phasic GABAergic transmission shifted the net-E/I ratio towards inhibition (Fig. 5). And vice versa, although baclofen via presynaptic GABA_B_Rs inhibited phasic GABA release, in parallel, it activated postsynaptic GABA_B_Rs shifting the net-E/I ratio towards inhibition as well. Thus presynaptic and postsynaptic GABA_B_Rs operate in concert to prevent hyperactivity of mPFC in WT cortex independently on the extracellular GABA level. This is however not the case in the Tsc2^+/-^ mPFC, where GABA_B_R activation strongly suppressed phasic GABA release, and the lack of postsynaptic GABA_B_R-mediated inhibition ultimately resulted in an overall excitatory baclofen action on the network (Fig. 5). These results obtain particular relevance if we consider that modulation of GABA_B_Rs has been contemplated as a potential treatment for ASD symptoms in both mice and humans. The overall excitatory effect of baclofen on the network activity observed in this study should be taken into consideration in case of therapeutic application of drugs acting on GABA_B_Rs.
